# Synthesis of Mesoporous and Hollow SiO_2_@ Eu(TTA)_3_phen with Enhanced Fluorescence Properties

**DOI:** 10.3390/ma16134501

**Published:** 2023-06-21

**Authors:** Zhiheng Wang, Xiaoli Hu, Yinqi Yang, Wei Wang, Yao Wang, Xuezhong Gong, Caiyun Geng, Jianguo Tang

**Affiliations:** Institute of Hybrid Materials, National Center of International Research for Hybrid Materials Technology, National Base of International Science & Technology Cooperation, College of Materials Science and Engineering, Qingdao University, Qingdao 266071, China; a1191783491@gmail.com (Z.W.); 2021020497@qdu.edu.cn (X.H.); yangyingqi060@gmail.com (Y.Y.); wangyao72@qdu.edu.cn (Y.W.); xzgong@qdu.edu.cn (X.G.); gengcy4@gmail.com (C.G.)

**Keywords:** silica, Eu(TTA)_3_phen, fluorescence

## Abstract

Lanthanide ions are extensively utilized in optoelectronic materials, owing to their narrow emission bandwidth, prolonged lifetime, and elevated fluorescence quantum yield. Inorganic non-metallic materials commonly serve as host matrices for lanthanide complexes, posing noteworthy challenges regarding loading quantity and fluorescence performance stability post-loading. In this investigation, an enhanced Stöber method was employed to synthesize mesoporous hollow silica, and diverse forms of SiO_2_@Eu(TTA)_3_phen (S@Eu) were successfully prepared. Transmission electron microscopy (TEM), energy-dispersive X-ray spectroscopy (EDS), Fourier-transform infrared (FTIR) spectroscopy, and X-ray photoelectron spectroscopy (XPS) outcomes revealed the effective binding of silica with Eu(TTA)_3_phen through both physical adsorption and chemical bonding. This includes the formation of Si-O-C bonds between silica and the ligand, as well as Si-O-Eu bonds between silica and europium ions. Fluorescence tests demonstrated that the mesoporous SiO_2_@Eu(TTA)_3_phen(MS@Eu) composite exhibited the highest fluorescence intensity among the three structured silica composites, with a notable enhancement of 46.60% compared to the normal SiO_2_@Eu(TTA)_3_phen composite. The Brunauer–Emmett–Teller (BET) analysis indicated that the specific surface area plays a crucial role in influencing the fluorescence intensity of SiO_2_@Eu(TTA)_3_phen, whereby the prepared mesoporous hollow silica further elevated the fluorescence intensity by 61.49%. Moreover, SiO_2_@Eu(TTA)_3_phen demonstrated 11.11% greater cyclic stability, heightened thermal stability, and enhanced alkaline resistance relative to SiO_2_@Eu(TTA)_3_phen.

## 1. Introduction

Rare-earth metal elements possess a distinctive electronic structure characterized by the 4f subshell, which bestows upon them exceptional optical, electrical, and magnetic properties [1,2,3]. They have found widespread utilization across various industries, including the military, metallurgy, petrochemicals, electronics, glass and ceramics, and agriculture. In particular, rare-earth metals have gained prominence in high-tech sectors such as new materials and electronics, and they are recognized as strategic resources by numerous nations worldwide [4]. Among the diverse avenues of research into functional rare-earth materials, the investigation of luminescent rare-earth materials holds paramount importance [5,6,7,8,9]. Owing to the limited molar absorptivity (less than 10 L mol^−1^ cm^−1^) of trivalent lanthanide ions and the forbidden f-f transitions, only a minute fraction of radiation directly excites the 4f level of rare-earth ions, resulting in feeble luminescence. The effective solution lies in the incorporation of rare-earth complexes into inorganic materials. In this approach, organic ligands possessing strong absorption bands in the ultraviolet (UV) region can capture more light energy than the lanthanide ions themselves. Subsequently, the excited energy is transferred from the organic ligands to the lanthanide ions through intramolecular energy transfer, enabling the sensitization of rare-earth ions to emit stronger light compared to pure lanthanide ions [10]. The rigid lattice structure of inorganic materials provides a stable microenvironment for luminescent lanthanide ions. The incorporation of lanthanide complexes into inorganic materials offers several advantages, including large Stoke shifts, narrow emission spectra, prolonged lifetimes, and high photochemical stability [11,12,13,14,15,16,17]. These merits endow lanthanide-doped inorganic materials with significant advantages across numerous applications, laying a solid foundation for modern technologies such as lighting, photon communication, battery devices, and bioimaging [18,19,20,21].

Silicon dioxide, a vital inorganic compound, possesses remarkable chemical stability and hardness. It features a compact and rigid crystal structure, along with exceptional insulation performance and biocompatibility. Silicon dioxide finds extensive application in the fabrication of optical glass [22], semiconductor devices [23], catalysts [24], coatings [25,26], ceramics [27], rubber [28], and plastics [29]. Moreover, it is widely employed in the medical [30], cosmetic [31], food [32], and environmental protection fields [33]. Within the realm of materials research, mesoporous silica and hollow silica have emerged as prominent topics of exploration. Mesoporous silica refers to a porous material characterized by a regular pore structure, with pore sizes ranging from 2 to 50 nanometers [34,35]. Several methods, such as the sol–gel method, hydrothermal method, and electrochemical method, can be employed for synthesizing mesoporous silica. Due to its adjustable pore structure, substantial specific surface area, controllable pore size, and high surface activity, mesoporous silica finds versatile applications in adsorption, catalysis, separation, sensing, and various other fields [36]. On the other hand, hollow silica is a material with a hollow internal structure and a solid silicon oxide exterior. Unlike mesoporous silica, there are relatively fewer methods for synthesizing hollow silica, primarily including the template method, membrane reaction method, and hydrothermal method [26,37]. Hollow silica possesses advantages such as a high specific surface area, hollow structure, and controllable pore size, making it widely applicable in areas such as drug delivery, catalyst carriers, and sensors. The common approaches for synthesizing mesoporous silica and hollow silica mainly involve the employment of either the hard template method or the soft template method [38,39]. The hard template method offers advantages such as precise preparation, controllable pore size, and excellent pore connectivity [40]. However, it is associated with drawbacks such as complex preparation procedures, the necessity for template and deposition material preparation, and the potential for pore channel deformation or shrinkage following template removal. In contrast, the soft template method is characterized by a simpler preparation process, a wider range of pore sizes, and favorable pore connectivity [41]. Nonetheless, it exhibits disadvantages such as lower pore size accuracy and uneven pore size distribution. A template-free method for synthesizing hollow silica presents advantages such as simplicity, controllable pore size, large-scale production, and minimal pollution, thus holding great potential for further exploration [42,43,44,45,46,47].

Silica-based rare-earth materials exhibit a wide range of applications [48,49,50,51,52,53,54]. Notably, Tin-gyun Wang et al. [55] recently presented a novel approach for fabricating transparent Ce/Tb co-doped yttrium pyrosilicate (YPS) nanocrystal silica fibers using the C_2_ laser heating method. By embedding Ce^3+^ and Tb^3+^ ions, they achieved an impressive energy transfer efficiency of up to 59.87% and improved fluorescence performance. This advancement positions the material as a promising candidate for scintillators, fiber lasers, and phosphors. In a separate investigation conducted by Yujuan He et al. [56], films of europium-doped yttrium orthovanadate (YVO_4_:Eu) and hollow silica nanoparticles (HSNPs) were prepared and applied onto a glass cover of a polycrystalline silicon solar cell. The resulting film demonstrated downshifting and anti-reflection capabilities, effectively capturing ultraviolet photons and minimizing reflection. This innovation renders the material suitable for integration into solar cell modules.

**Figure 1:**
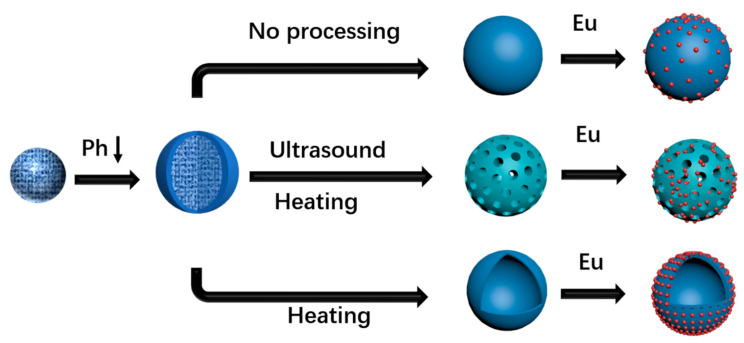
Preparation process of SiO_2_@Eu(TTA)_3_phen with different structures.

Previous research on the combination of rare-earth metals and silica has predominantly focused on silica fibers or thin films [57], while studies exploring the combination of distinct structures of spherical silica and rare-earth complexes have been scarce. This study aimed to address this gap by preparing silica spheres with varying degrees of internal and external shrinkage. Through ultrasonic and heating treatments, silica spheres with diverse morphologies, including solid, mesoporous, and hollow structures, were successfully obtained. The rare-earth complex Eu(TTA)_3_phen was subsequently adsorbed onto the surfaces of these silica spheres, resulting in impressive fluorescence performance (Figure 1). A comparison of the fluorescence performance with other structures of SiO_2_@Eu(TTA)_3_phen revealed that mesoporous SiO_2_@Eu(TTA)_3_phen exhibited superior performance and demonstrated significant potential in battery devices. Furthermore, the fluorescence intensity variations and cyclic stability of SiO_2_@Eu(TTA)_3_phen and Eu(TTA)_3_phen were evaluated under different environmental temperatures and pH values, with SiO_2_@Eu(TTA)_3_phen demonstrating excellent performance.

## 2. Materials and Methods

### 2.1. Materials

Ethanol, tetramethylammonium hydroxide (TMAH), and tetraethyl orthosilicate (TEOS) were acquired from Beijing Chemical Corporation. Ammonium hydroxide (AR grade) and 1,10-phenanthroline (phen) were obtained from Sinopharm Chemical Reagent Co., Ltd. (Shanghai, China). Europium oxide was sourced from Darui Company (Shanghai, China). Trifluoroacetylacetone (TTA) was procured from Shanghai McLean Biochemical Co., Ltd. Solvent-grade ethanol was utilized directly without further treatment. Ethyl orthosilicate underwent distillation prior to use. High-purity water (Pall Purelab Plus) with a resistivity of 18 M Ω/cm was employed in all experimental procedures.

### 2.2. Preparation of Silica Spheres

The modified Stöber method [58] was employed for the preparation process. To synthesize silica spheres, a solution containing 50 mL of ethanol, 1.0 mL of water, and 1.0 mL of 0.1 M tetramethylammonium hydroxide (TMAH) was introduced into an oil bath placed within a three-necked flask. Subsequently, 1.25 mL of tetraethyl orthosilicate (TEOS) was added to the solution, which was stirred at a rate of 800 rpm for a duration of three hours. Afterward, the mixture underwent centrifugation at 10,000 rpm for a period of 20 min, allowing for the collection of the solid precipitates.

### 2.3. Preparation of Hollow Silica

The collected solid precipitates were introduced into 50 mL of boiling water containing an appropriate amount of TMAH and subjected to an etching process for a duration of three hours. Following etching, the mixture underwent centrifugation at 10,000 rpm, allowing for the collection of the resulting solid materials.

### 2.4. Preparation of Mesoporous Silica

The collected solid precipitates were washed twice and subsequently added to hot water at a temperature of 90 °C, along with an appropriate amount of TMAH, for etching over a period of three hours. Following the etching process, the mixture underwent centrifugation at 10,000 rpm, allowing for the collection of the resulting solid material.

### 2.5. Preparation of Mesoporous Hollow SiO_2_

The collected silica solids were introduced into an ethanol solution of TMAH with a pH of 10.8 and subjected to sonication for a duration of 30 min. Subsequently, the mixture was centrifuged, and the resulting solid precipitates were collected. These solids were further subjected to heating and stirring in boiling water for a period of 3 h. After undergoing centrifugation once again, the solids were sonicated and washed twice using ethanol.

### 2.6. Preparation of SiO_2_@Eu(TTA)_3_phen with Four Morphologies

In order to prepare silica spheres with different morphologies, a mixture of 9.5 mL of ethanol and 0.1 mL of 0.1 M phenanthroline (phen) solution was added to the previously obtained silicon dioxide spheres with varying morphologies. The mixture was then subjected to sonication for a duration of 30 min. Subsequently, 0.1 mL of 0.1 M EuCl_3_ and 0.3 mL of 0.1 M thenoyltrifluoroacetone (TTA) were added to the mixture, which was stirred for three hours at room temperature. After undergoing centrifugation, the solid precipitates were collected and washed twice using ethanol.

### 2.7. Preparation of SiO_2_@Eu(TTA)_3_phen with Three Morphologies

In a three-necked flask immersed in an oil bath, mix 50 mL of ethanol, 1.0 mL of water, and 1.0 mL of TMAH (0.1 M). Add 5 mL of TEOS to the mixture and stir at 800 rpm for 3 h. Centrifuge the mixture at 10,000 rpm for 20 min and collect the solid. Mix the prepared silica spheres with 9.5 mL of ethanol and 0.1 mL of phen (0.1 M) solution. Ultrasonicate the mixture for 30 min. Add 0.1 mL of EuCl_3_ (0.1 M), 0.3 mL of TTA (0.1 M), and 0.35 mL of NH_4_OH (0.1 M) to the solution and stir at room temperature for 2.5 h. Collect the solid after centrifugation. Repeat this step three times to prepare three sets of samples.

Dilute the collected S@Eu and Eu solids with 5 mL of ethanol. Divide the mixture into five groups and stir each group at 10, 30, 50, 70, and 90 degrees Celsius for 2 h. After washing, dissolve the solids in a 5 mL ethanol solution.

Divide the collected S@Eu and Eu solids into five groups and immerse them in ethanol solutions with pH values of 3, 5, 7, 9, and 11. Adjust the pH by adding HCl or TMAH and stir for 2 h. After washing, dissolve the solids in a 5 mL ethanol solution.

Dilute the collected S@Eu and Eu solids with 5 mL of ethanol. Centrifuge and sonicate each group 1, 2, 3, 4, and 5 times. Collect the solids and dissolve them in a 5 mL ethanol solution. Repeat this process three times to prepare three sets of samples.

### 2.8. Characterizations

Transmission electron microscopy (TEM): TEM tests were performed using the Tecnai G2 F20 S-TWIN field-emission transmission electron microscope (FEI America). A small amount of prepared sample ethanol solution was taken using a pipette and dropped onto a carbon-film-coated copper grid. The grid was then dried under a drying lamp. The TEM images were obtained to examine the morphology and structure of the samples. Fourier-transform infrared (FTIR) spectroscopy: FTIR spectra were recorded using the Nicolet 670 Fourier-transform infrared spectrophotometer. First, the background infrared spectrum was recorded by adding ethanol to the sample chamber. After cleaning the sample chamber, the prepared sample ethanol solution was added, and the infrared spectrum of the sample was measured. The FTIR spectra provided information about the functional groups present in the samples. X-ray photoelectron spectroscopy (XPS): XPS analysis was performed using the Thermo Scientific Escalab 250Xi. The sample, after thorough drying, was affixed to a conductive adhesive and tested. XPS provided information about the elemental composition and chemical states of the elements present in the samples. Brunauer–Emmett–Teller (BET) surface area measurement: BET surface area measurements were conducted to determine the specific surface area of the samples. Approximately 100 mg of the sample was placed in a test tube and subjected to vacuuming for 10 h to remove adsorbed gas. After vacuuming, the sample was cooled to room temperature and backfilled with nitrogen gas. The weight of the sample tube was recorded, and the sample was weighed using the decrement method. The analysis station was used to measure the sample and obtain the BET surface area. energy-dispersive spectrometry (EDS): EDS analysis was performed using the FEI QUANTA 250 FEG. The samples were characterized at an accelerating voltage of 15 kV. EDS provided information about the elemental composition of the samples.

## 3. Results and Discussion

### 3.1. Preparation Process Analysis

In the Stöber method, the designated silicon source for the preparation of SiO_2_ is known as tetraethyl orthosilicate (TEOS). This compound undergoes hydrolysis to yield silanol groups (-Si-OH), effectively replacing the ethoxy groups (Si-OR). The pace of this reaction is influenced by the extent of ethoxy group conversion into silanol groups. In the typical Stöber synthesis, a catalyst such as ammonia solution is employed to expedite the hydrolysis and condensation reactions. The presence of OH^−^ ions in the ammonia solution enhances their efficacy as nucleophiles. Both hydrolysis and condensation reactions entail nucleophilic substitution, yet the condensation reaction proceeds at a considerably swifter pace compared to hydrolysis. This is attributed to the propensity of silanol groups to release protons more readily than water molecules, resulting in an augmented positive charge density around the silicon atom. Consequently, nucleophilic substitution becomes more favorable. Consequently, silanol monomers display a greater inclination to connect with larger silica clusters, as opposed to other monomers or smaller oligomers.

**Figure 2:**
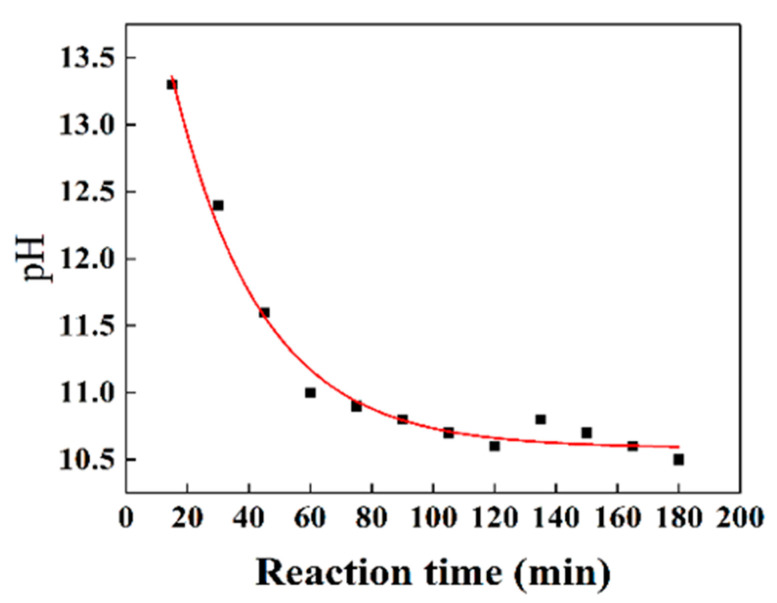
Time-dependent curve of solution pH during the preparation of SiO_2_.

By manipulating the TMAH content within the silica spheres, three distinct types of silica particles were synthesized, each exhibiting solid, mesoporous, and hollow structures. TMAH, functioning as a catalyst for silica preparation, induces a rapid decline in pH throughout the reaction process, thereby influencing the rates of hydrolysis and condensation of TEOS. During the initial stages of the reaction, both hydrolysis and condensation occur at an accelerated pace, giving rise to a less densely consolidated silica structure that is susceptible to etching. However, in the subsequent stages, these processes decelerate, resulting in the formation of highly condensed silica that resists etching. Figure 2 provides a depiction of the pH variation over time in a reaction solution catalyzed by 2.0 mM TMAH. Within the first hour, the pH of the solution experiences a sharp decline from 13.3 to 11.1, gradually settling at 10.5 over the subsequent two hours. The pH fluctuations exert a direct influence on the hydrolysis and condensation reactions of TEOS facilitated by TMAH, which exhibit heightened reactivity during the initial hour but subsequently decelerate. This deceleration can be attributed to the pronounced drop in pH as the reaction progresses. These distinct stages promote the formation of loosely and tightly aggregated silica gel networks, respectively.

### 3.2. Morphological Analysis of Silica-Doped Europium Complexes

Through the application of ultrasound and pH adjustment, it became possible to manipulate the concentration and spatial arrangement of tetramethylammonium hydroxide (TMAH) within silica spheres characterized by varying degrees of inner and outer condensation. By subjecting these spheres to alkaline boiling conditions, distinct outcomes were achieved based on the TMAH content. Spheres harboring a greater quantity of TMAH internally underwent etching, resulting in the formation of hollow silica spheres. On the other hand, spheres with a lower and more dispersed concentration of TMAH yielded mesoporous silica spheres through the etching process. Meanwhile, solid silica spheres were obtained from those containing an exceedingly minimal amount of TMAH, even after the etching procedure.

**Figure 3:**
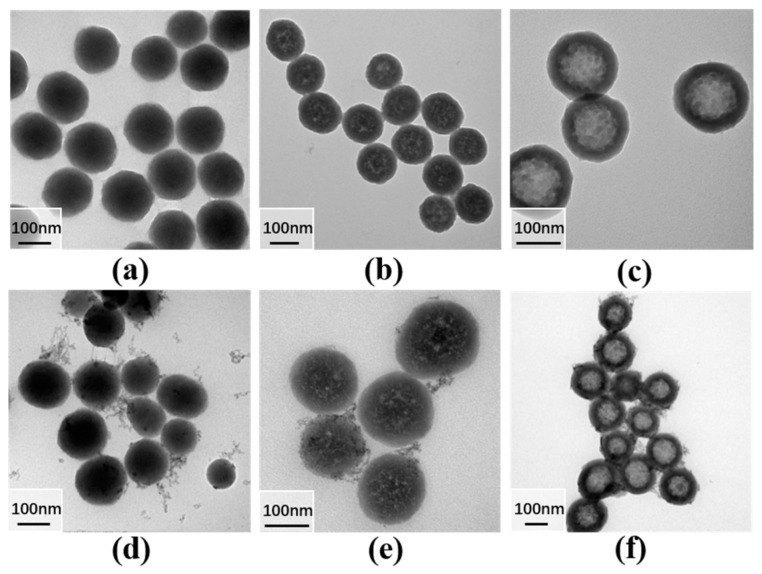
TEM images of silica with different morphologies: (**a**) silica; (**b**) mesoporous silica; (**c**) hollow silica; (**d**) S@Eu; (**e**) MS@Eu; (**f**) HS@Eu.

Figure 3a–c showcase transmission electron microscopy (TEM) images of solid, mesoporous, and hollow SiO_2_ particles, respectively. Additionally, Figure 3d–f present TEM images of solid S@Eu, mesoporous MS@Eu, and hollow SiO_2_@Eu(TTA)_3_phen (HS@Eu) complexes, respectively. The synthesized SiO_2_ particles exhibited an average diameter of 150 nm, with the hollow SiO_2_ possessing a shell thickness of approximately 27 nm. Notably, the morphologies of the complexes varied depending on their concentrations, as depicted in Figure S1. The images in Figure 3d–f vividly demonstrate the uniform dispersion of flocculent materials on the surface of SiO_2_ particles. Moreover, it can be observed that the flocculent materials exhibit greater density surrounding MS@Eu and HS@Eu, while fewer free flocculent materials are present near regular SiO_2_. This reaffirms the adsorption of flocculent materials onto SiO_2_, as confirmed by Figure 3.

**Figure 4:**
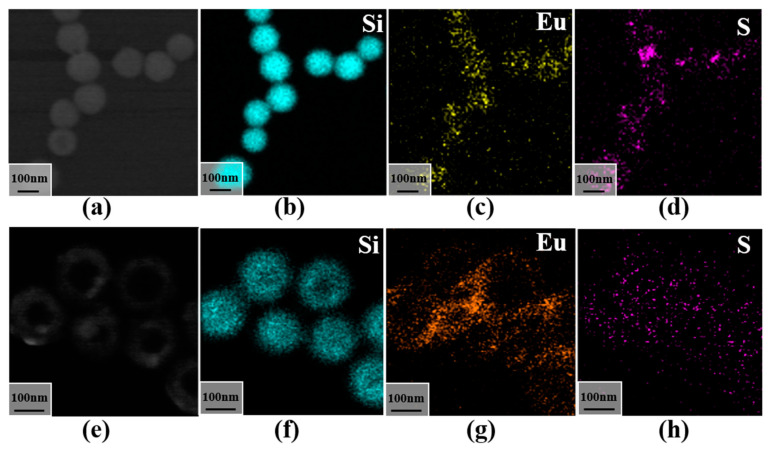
EDS mapping images of different morphologies of silica coated with Eu (TTA)_3_phen: (**a**) TEM image of MS@Eu; (**b**) Si elemental mapping of MS@Eu; (**c**) Eu elemental mapping of MS@Eu; (**d**) S elemental mapping of MS@Eu; (**e**) TEM image of HS@Eu; (**f**) Si elemental mapping of HS@Eu; (**g**) Eu elemental mapping of HS@Eu; (**h**) S elemental mapping of HS@Eu.

To further investigate the nature of the flocculent material—specifically, whether it corresponds to Eu(TTA)_3_phen—an energy-dispersive X-ray spectroscopy (EDS) mapping analysis was conducted. Figure 4a–c,f demonstrate the uneven distribution of the elements Eu and S on the mesoporous silica spheres, with the S derived from 2-thenoyltrifluoroacetone. Similarly, EDS mapping analysis was performed on the hollow silica spheres, as presented in Figure 4e–h. The distinct characteristic of the hollow silica spheres is the presence of a lower concentration of Si in the central region and a higher concentration in the outer layer, which is consistent with the findings from the TEM examination. The S and Eu are well dispersed on the outer layer of the silica. Based on the EDS spectrum and TEM results obtained from the hollow silica analysis, it can be concluded that the flocculent material corresponds to Eu(TTA)_3_phen.

### 3.3. Analysis of the Binding Mechanism of S@Eu

**Figure 5:**
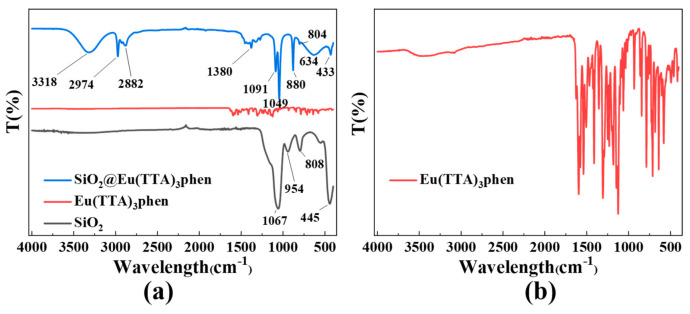
(**a**) FTIR of Eu(TTA)_3_phen, SiO_2_, and MS@Eu; (**b**) FTIR of Eu(TTA)_3_phen.

Figure 5 illustrates the FTIR spectrum. Within the silica infrared spectrum, the peaks at 808 cm^−1^ and 445 cm^−1^ correspond to the symmetric stretching vibration of Si-O bonds, while the peak at 954 cm^−1^ corresponds to the bending vibration of Si-OH bonds. Notably, there is a prominent and broad peak at 1067 cm^−1^, representing the antisymmetric stretching vibration of Si-O-Si bonds. In the infrared spectrum of Eu(TTA)_3_phen, numerous small peaks in the range of 500–1700 cm^−1^ arise from the internal vibrations of Eu(TTA)_3_phen molecules. For instance, the CF_3_ stretching vibration occurs within the 1120–1350 cm^−1^ range, while the phenyl ring exhibits aromatic ring vibration typically observed at 1600–1500 cm^−1^. Additionally, the carbonyl group generates a carbonyl stretching vibration at 1700–1600 cm^−1^, and the aromatic amine group induces an amino stretching vibration at 3400–3200 cm^−1^. Regarding S@Eu, the absence of the peak at 954 cm^−1^ can be attributed to the reaction between the Si-OH groups on the silica surface and TTA, resulting in the formation of hydrogen bonds. Furthermore, the disappearance of the peak at 808 cm^−1^ and the emergence of the peaks at 880 cm^−1^ and 634 cm^−1^ can be attributed to the formation of Si-O-Eu bonds between Eu(TTA)_3_phen and the silica surface, along with the formation of Eu-N bonds resulting from the coordination between Eu and phen during the complex formation. Additional peaks observed include 2882 cm^−1^ and 2974 cm^−1^, corresponding to the symmetric and antisymmetric stretching vibrations of CH_3_ in Eu(TTA)_3_phen, respectively. The peak at 3318 cm^−1^ represents the stretching vibration of N-H, derived from the residual amino group of ammonia.

**Figure 6:**
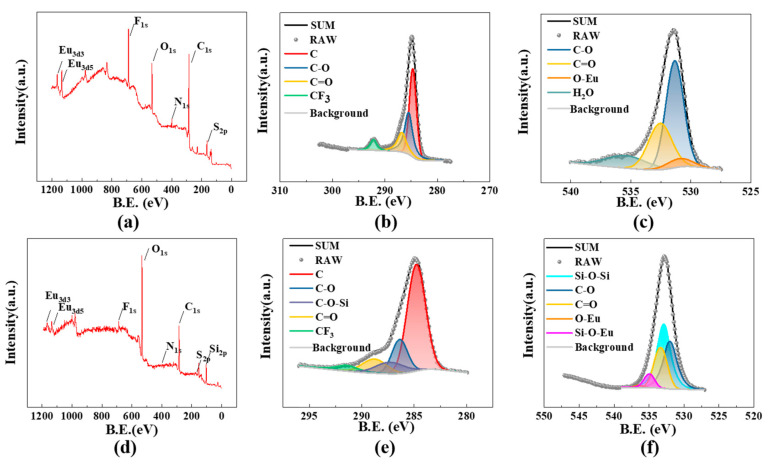
XPS images of silica with different morphologies: (**a**) XPS for Eu(TTA)_3_phen; (**b**) XPS of C1s for Eu(TTA)_3_phen; (**c**) XPS of O1s for Eu(TTA)_3_phen; (**d**) XPS for S@Eu; (**e**) XPS of C1s for S@Eu; (**f**) XPS of O1s for S@Eu.

The C 1s XPS analysis, depicted in Figure 6b,e, provides valuable insights. It reveals the presence of stable CF_3_ peaks at approximately 292 eV for both Eu(TTA)_3_phen and Si@Eu, originating from the TTA ligand. Furthermore, C-O and C=O peaks are observed at 286 eV and 288 eV, respectively. Notably, Si@Eu exhibits an additional peak at 287 eV, which arises from the reaction between the C=O bond in the TTA ligand and the -OH groups on the surface of the silica. This reaction leads to the formation of a C-O-Si bond. The fitting results of the oxygen peak, displayed in Figure 6c,f, indicate that the binding energy of the O-Eu bond in Eu(TTA)_3_phen is measured at 530 eV. In comparison, S@Eu not only forms bonds with the O atoms in the ligand but also establishes a Si-O-Eu bond with the O atoms on the surface of the silica, resulting in a peak located at 535 eV. The abundance of Si-O-Si bonds indicates that the primary component of S@Eu is still silica. Moreover, infrared analysis confirms the coexistence of both physical adsorption and chemical bonding between the silica and Eu(TTA)_3_phen.

### 3.4. Analysis of the Fluorescence Properties of S@Eu

**Figure 7:**
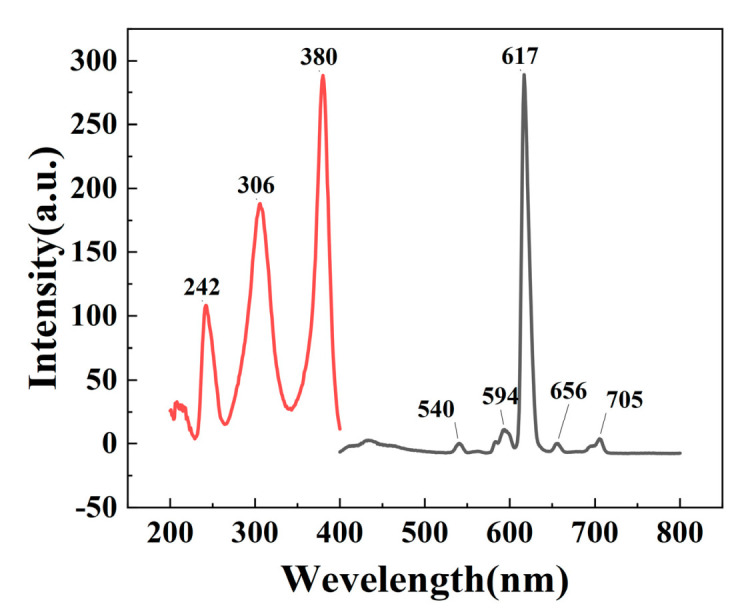
Fluorescence excitation and fluorescence emission diagram of Eu(TTA)_3_phen.

The excitation peak is shown in the left panel of Figure 7, with the normal f-f absorption of Eu^3+^ occurring around 380 nm, the absorption of TTA and phen ligands appearing around 306 nm, and the π-π* transition of TTA at around 240 nm. Fluorescence emission spectra were measured with the strongest excitation at 380 nm, as shown in the right panel of Figure 7, exhibiting a characteristic emission peak at 617 nm, which corresponds to the ^5^D_0_→^7^F_2_ (617 nm) transition of Eu^3+^. The other two peaks at 594 nm and 656 nm correspond to the ^5^D_0_→^7^F_1_ and ^5^D_0_→^7^F_3_ transitions, respectively. The emission peak at 617 nm is the strongest, while that at 656 nm is the weakest, indicating the characteristic fluorescence emission of Eu^3+^. The position of the peak is essentially the same as previously reported [59].

**Figure 8:**
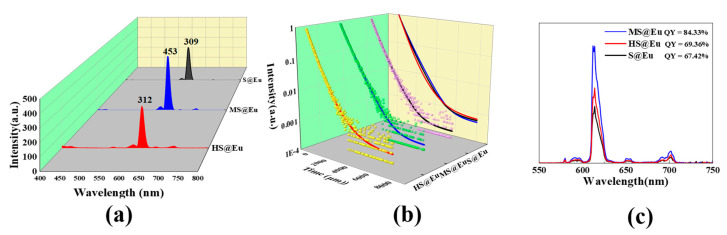
(**a**) Fluorescence intensity; (**b**) fluorescence lifetime fitting curve; (**c**) quantum yield.

Figure 8a presents the fluorescence intensity and lifetime measurements. It can be observed that MS@Eu demonstrates the highest fluorescence intensity, reaching 453. Following that, HS@Eu exhibits an intensity of 312, while S@Eu shows the weakest fluorescence intensity, at 309. The comparison of the fluorescence lifetimes and fitting curves is depicted in Figure 8b, with the corresponding fitting data provided in Table 1. Among the samples, MS@Eu showcases the most favorable fluorescence lifetime performance, with a lifetime of 557 μs. S@Eu exhibits a fluorescence lifetime of 549 μs, while HS@Eu displays a fluorescence lifetime of 460 μs. Furthermore, Figure 8c illustrates the quantum yield data. MS@Eu demonstrates the highest quantum yield, reaching 84.33%. In comparison, HS@Eu and S@Eu exhibit quantum yields of 69.36% and 67.42%, respectively. As a result, it can be concluded that MS@Eu exhibits the most superior overall fluorescence performance among the samples.

**Figure 9:**
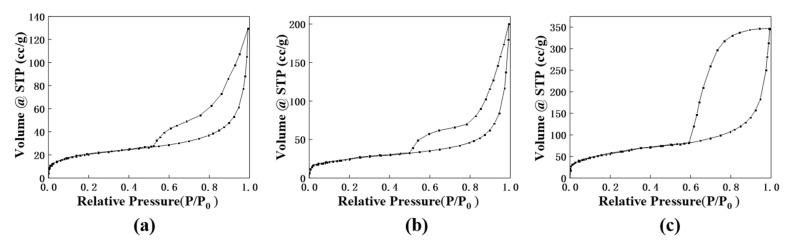
N_2_ adsorption-desorption isotherms: (**a**) SiO_2_; (**b**) hollow SiO_2_; (**c**) mesoporous SiO_2_.

The variation in fluorescence intensity among the three structures can be attributed to the specific surface area. The nitrogen adsorption-desorption isotherms for the three structures are presented in Figure 9a–c. Upon analysis, it was found that mesoporous SiO_2_ possesses the highest BET specific surface area of 261 m^2^/g, followed by hollow SiO_2_ with a value of 200 m^2^/g, and SiO_2_ with a value of 153 m^2^/g. This indicates that mesoporous SiO_2_ exhibits the largest surface area among the three structures. It is noteworthy that MS@Eu, which is composed of mesoporous SiO_2_, demonstrates the strongest fluorescence intensity. This observation aligns with the expectations based on the test results, as a higher specific surface area generally facilitates enhanced fluorescence intensity.

**Figure 10:**
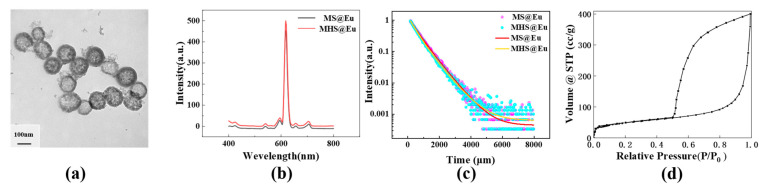
(**a**) TEM images of HMS@Eu; (**b**) fluorescence intensity of HMS@Eu and MS@Eu; (**c**) fluorescence lifetimes of HMS@Eu and MS@Eu; (**d**) N_2_ adsorption-desorption isotherms of MHS.

Mesoporous hollow silica (MHS) was effectively synthesized by manipulating the duration of the ultrasonic treatment and adjusting the pH of the solution, utilizing the principles of mesoporous silica and hollow silica. As depicted in Figure 10b,c, MHS@Eu exhibits a higher fluorescence intensity compared to MS@Eu, albeit with a slightly lower fluorescence lifetime of 481 μs. Additionally, the quantum yield of MHS@Eu was measured to be 85.65%. Overall, MHS showcases superior fluorescence performance, along with a larger specific surface area, as illustrated in the figures.

**Figure 11:**
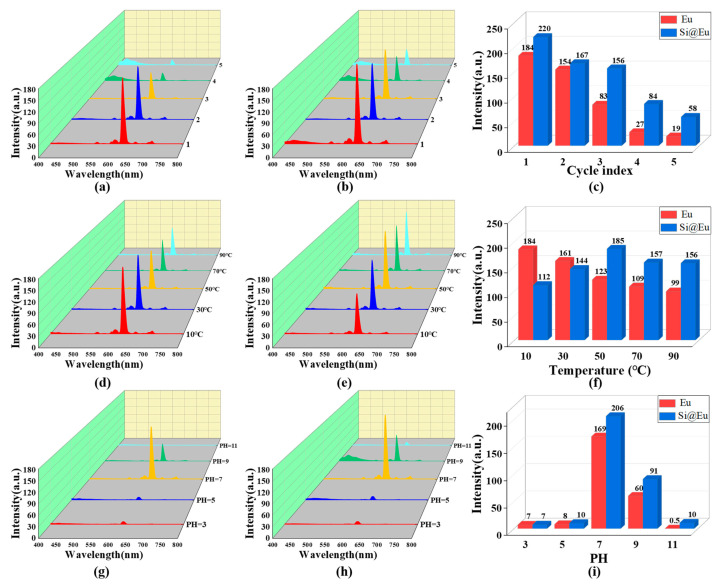
(**a**) Fluorescence intensity of Eu(TTA)_3_phen varies with the number of cycles. (**b**) Fluorescence intensity of S@Eu varies with the number of cycles. (**c**) Cyclic stability. (**d**) Fluorescence intensity of Eu(TTA)_3_phen varies with temperature. (**e**) Fluorescence intensity of S@Eu varies with temperature. (**f**) High-temperature resistance. (**g**) Fluorescence intensity of Eu(TTA)_3_phen varies with pH. (**h**) Fluorescence intensity of S@Eu varies with pH. (**i**) Acid–alkali resistance.

The cyclic stability test results, as depicted in Figure 11a–c, reveal a significant decline in the fluorescence intensity of Eu(TTA)_3_phen and S@Eu with increasing centrifugation–sonication cycles. Eventually, the fluorescence intensity of both materials reaches a very low level. However, due to the binding of Eu(TTA)_3_phen to the surface of silica with a relatively large particle size, S@Eu manages to maintain a relatively favorable fluorescence intensity even after multiple cycles. The fluorescence intensity variations with temperature, as displayed in Figure 11d–f, exhibit distinct patterns. As the temperature rises, the fluorescence intensity of Eu(TTA)_3_phen experiences a rapid decrease. Conversely, the fluorescence intensity of S@Eu initially increases and then decreases, reaching its peak between 30 °C and 50 °C. In comparison to pure Eu(TTA)_3_phen, S@Eu demonstrates improved fluorescence intensity at high temperatures, owing to the exceptional heat resistance of silica. The acid–base resistance test outcomes, as demonstrated in Figure 11g–i, indicate poor acid resistance for both Eu(TTA)_3_phen and S@Eu, with the fluorescence intensity reaching its lowest point at pH 5. However, when compared to Eu(TTA)_3_phen, S@Eu exhibits better alkali resistance. This can be attributed to the ability of silica to bind to certain −OH groups and mitigate the adverse effects of strong alkaline conditions on the fluorescence performance of Eu(TTA)_3_phen.

## 4. Conclusions

Hollow, mesoporous silica was prepared using an improved Stöber method and different etching conditions; subsequently, different structures of S@Eu were prepared. The binding between the silica and rare-earth complexes was confirmed by TEM and EDS tests. The binding mechanism between the two was analyzed through XPS and infrared spectroscopy, revealing that the binding between silica and rare-earth complexes is not only physical adsorption but also involves bonds such as Si-O-Eu and Si-O-C. Fluorescence intensity and lifetime tests showed that the fluorescence performance of silica-doped europium complexes was effectively improved, with mesoporous silica showing the best performance. Based on the BET test, it was found that specific surface area was the main factor affecting the fluorescence performance of the products. MHS@Eu was prepared, which further improved the fluorescence intensity. Finally, the cycling stability, high-temperature resistance, and acid–base resistance of Eu(TTA)_3_phen and S@Eu were compared, with S@Eu showing excellent resistance to high temperatures and bases, as well as excellent cycling stability. This research has broad potential applications in fluorescence probes and other fields.

## Figures and Tables

**Table 1:** Fluorescence lifetime fitting parameters.

Sample	y0	A1	t1	A2	t2	τ
HS@Eu	6.04 × 10^−4^	1.23	460,387.78	0.10	950,775.77	460 μs
Ms@Eu	4.71 × 10^−4^	0.51	391,854.01	0.77	712,052.92	557 μs
S@Eu	5.47 × 10^−4^	0.85	436,486.49	0.47	730,546.66	549 μs

## Data Availability

The data that support the findings of this study are available from the corresponding author upon reasonable request.

## Supplementary Materials

The following supporting information can be downloaded at: https://www.mdpi.com/article/10.3390/ma16134501/s1, Figure S1 Different concentrations of Eu(TTA)_3_phen: (a) 0.00001 mol/L; (b) 0.0001 mol/L; (c) 0.001 mol/L; (d) 0.01 mol/L.
