# Supplementary figures and images for: Synthesis of Mesoporous and Hollow SiO2@ Eu(TTA)3phen with Enhanced Fluorescence Properties
Source: Materials (Basel). 2023 Jun 21;16(13):4501. doi: 10.3390/ma16134501 (PMC10342752; doi:10.3390/ma16134501)

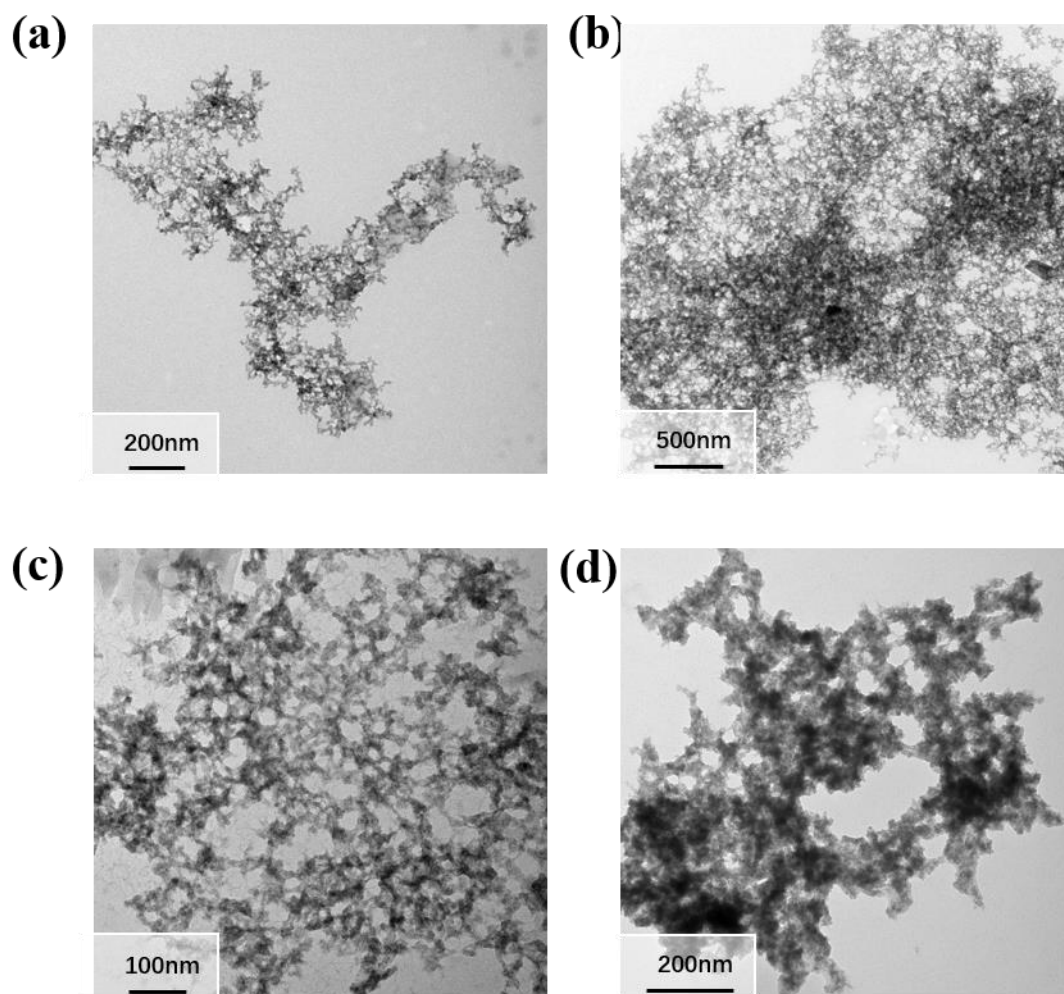

**Figure S1** Different concentrations of  $\text{Eu}(\text{TTA})_3\text{phen}$  : (a) 0.00001 mol/L; (b) 0.0001 mol/L; (c) 0.001 mol/L; (d) 0.01 mol/L.

Supplement: Supplementary file 1 [file materials-16-04501-s001.zip › materials-2413850-supplementary.pdf]
